# A bibliometric study of global trends in diabetes and gut flora research from 2011 to 2021

**DOI:** 10.3389/fendo.2022.990133

**Published:** 2022-10-21

**Authors:** Lu Zhang, Hongcai Zhang, Quan Xie, Shuai Xiong, Fengchen Jin, Fan Zhou, Hongjun Zhou, Jinhong Guo, Chuanbiao Wen, Biao Huang, Fei Yang, Yuanwei Dong, Ke Xu

**Affiliations:** ^1^ Chengdu University of Traditional Chinese Medicine, Chengdu, China; ^2^ Hospital of Chengdu University of Traditional Chinese Medicine, Chengdu, China; ^3^ North Sichuan Medical College, Nanchong, China; ^4^ Affiliated Hospital of Jiangxi University of Traditional Chinese Medicine, Nanchang, China; ^5^ Department of Oncology, Chongqing General Hospital, Chongqing, China

**Keywords:** diabetes, gut flora, bibliometric study, citespace, vosviewer

## Abstract

**Background and objectives:**

Diabetes mellitus is a serious metabolic disease that causes a serious economic burden worldwide. Gut flora is a major component of diabetes research, and the aim of this study was to understand the trends and major components of research related to diabetes and gut flora in the last 11 years.

**Methods:**

We searched the Web of Science Core Collection database for articles on diabetes and gut flora related research from 2011-2021 on July 2, 2022. The literature data were analyzed for country, institution, author, steward, journal, and highly cited literature using Citespace.5.8.R3 and Vosviewer1.6.17.

**Results:**

Finally 4834 articles that met the requirements were included. The overall trend of articles published in the last 11 years is increasing, and the trend of articles published after 2019 is increasing significantly. In total, 109 countries, 4820 institutions, and 23365 authors were involved in the field of research. The highest number of publications was 1262 articles from the United States, the institution with the most publications was the University of Copenhagen with 134 articles, and the author with the most publications was PATRICE D CANI with 52 articles.

**Conclusion:**

The number of studies related to diabetes and intestinal flora is increasing and more and more researchers are involved in this field. Intestinal flora provides a key research direction for the treatment of diabetes. In the future, gut flora will remain the focus of the diabetes field.

## Introduction

Chronic hyperglycemia is the primary symptom of diabetes mellitus, which is a broad name for a diverse metabolic condition of the body’s sugars. Currently, diabetes affects more than 425 million individuals globally (1). Type 1 diabetes, type 2 diabetes, and gestational diabetes are the three primary categories of diabetes (2). The greatest microbial system in the body is the flora of the intestines. Diabetes may result from changes in gut flora (3). One important aspect of human health is the kind of bacteria in the gut (4). Human health depends on the gut’s bacteria maintaining equilibrium (5). Individuals with diabetes have significantly different gut flora from healthy people (6). It is unclear how gut flora dysbiosis results in diabetes. It could be connected to the theories of fatty acids, bile acids, and endotoxins. For the prevention of diabetes, the research of diabetes based on gut flora is crucial.

Through statistical techniques, bibliometric analysis enables the quantitative investigation of trends in a topic (7). The method has been created and used in a variety of industries, including climate change (8), architecture (9), and. biology (10). There is, however, no bibliometric study of research on the relationship between gut flora and diabetes. Through a bibliometric examination of trends in research on diabetes and intestinal flora from 2011 to 2021, the goal of this study was to determine research priorities in this area. The study’s findings offer several research resources.

## Materials and methods

### Data source

**Figure 1:**
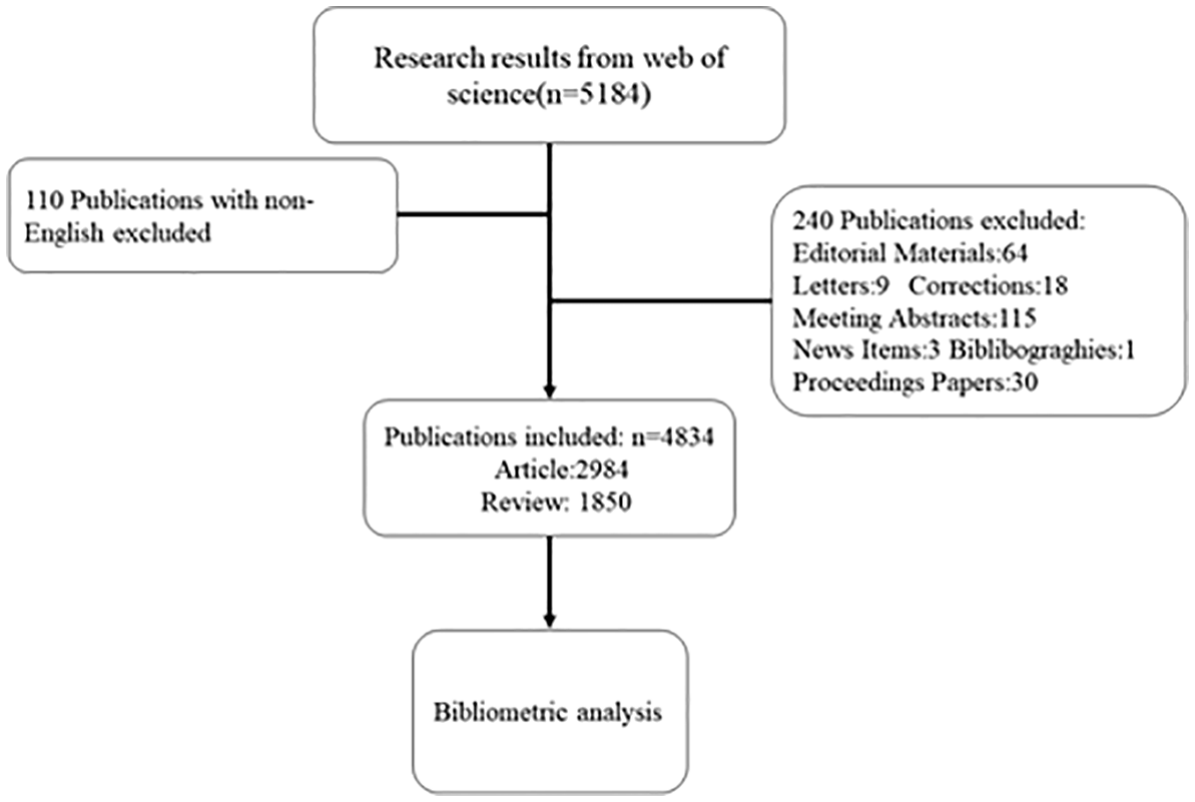
Retrieval process flowchart for the research.

The original data for this study were obtained from the Web of Science Core Collection database and were published from 2011-01-01 to 2021-12-31. article type was restricted to Article and Review only. 2 researchers conducted the search process independently, with the search formula: Topic=(“ diabetes mellitus” or “diabete*” or “diabetic*” or “diabetic mellitus”) AND Topic=(“gastrointestinal microbiome*” or “gut microbiome*” or “gut microflora” or “gut microbiota*” or “gastrointestinal flora” or “gut flora” or “gastrointestinal microbiota*” or “gastrointestinal microbial communit*” or “gastrointestinal microflora” or “gastric microbiome* “ or “intestinal microbiome*” or “intestinal microbiota*” or “ intestinal flora”). The search date was July 2, 2022, and the language was limited to: English. Excluding 350 non-compliant articles, 4834 valid articles were finally obtained as shown in **Figure 1**.

### Statistics and analysis

Choose the relevant, legitimate literature for the research and export it in the download* plain text format. To extract the year, nation, author, and keywords of the articles, import the raw data files into Citespace.5.8.R3. Then, use Microsoft Office Excel 2021 to count the years since publication and generate a table. Vosviewer 1.6.17 creates visual graphs while analyzing the raw literary data for journals and highly cited articles.

## Results

### Annual publication analysis

**Figure 2:**
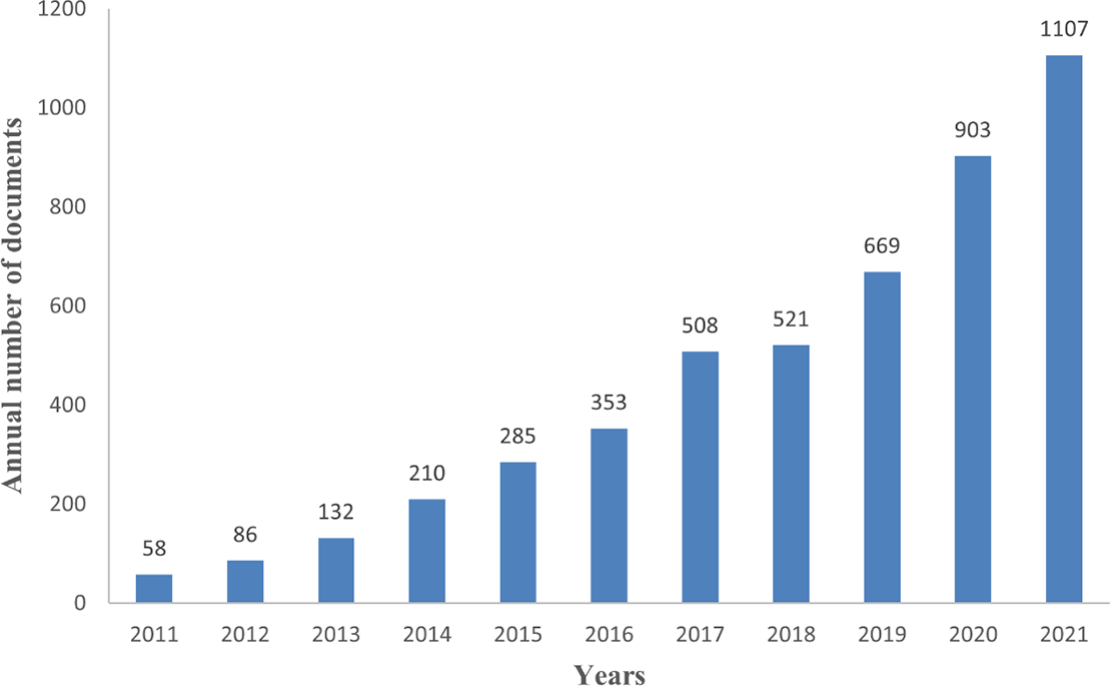
Trends in posting volume annually.

We used 4834 papers on diabetic gut flora research from 2011 to 2021 for this investigation. **Figure 2** illustrates the general upward trend in the number of papers on diabetic gut flora and the continued interest in this field of study. In 2017, there were more than 500 articles, while in 2021 there were a record-high 1107 pieces. Currently, research on the relationship between gut flora and diabetes is increasingly popular.

### Country/region analysis

The numbers show that from 2011 to 2021, research on gut flora and diabetes would be conducted in 109 different nations. **Table 1** lists the top 10 nations by the number of articles produced, with the United States ranking first with 1262. China and the United States are the two nations with more than 1000 articles each. Australia, Canada, France, Italy, and the United Kingdom are among the nations having 200 to 300 pieces each. Japan, Germany, and Spain are among the nations having less than 200 articles. The top 10 countries in Europe are Italy, the United Kingdom, France, Germany, and Spain with 1137 articles each, while the top 10 countries in Asia are the United States, Australia, and Japan with 1568 articles each. The top two countries in the Americas are the United States and Canada with 1505 articles each. The country’s capacity for cooperation increases with centrality.

**Table 1:** Top 10 countries/regions with the most publications.

Ranking	Countries/Area	Centrality	Year	Publications
1	USA	0.13	2011	1262
2	PEOPLES R CHINA.	0.03	2011	1174
3	ITALY.	0.03	2011	278
4	ENGLAND.	0.44	2011	266
5	CANADA.	0.00	2011	243
6	FRANCE.	0.03	2011	216
7	AUSTRALIA.	0.03	2011	201
8	JAPAN.	0.00	2011	193
9	GERMANY.	0.00	2011	191
10	SPAIN.	0.36	2011	186

### Analysis of major issuing institutions

**Figure 3:**
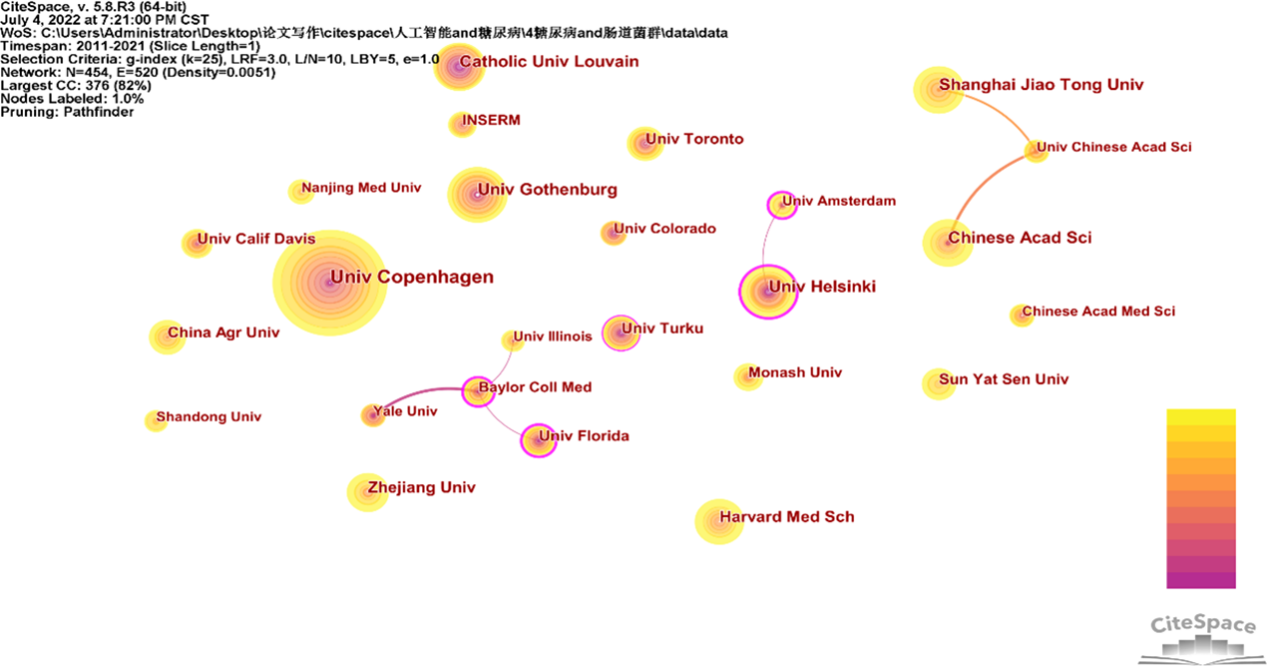
Cooperation network between major institutions.

Between 2011 and 2021, 4820 institutions were identified as participating in research on gut flora and diabetes. **Figure 3** depicts the collaboration partnerships between institutions that have more than 30 papers. University of Florida, Yale University, and University of Illinois are 3 universities with which Baylor College of Medicine has partnerships. Both the University of Helsinki and the University of Amsterdam work together. Shanghai Jiao Tong University and the Chinese Academy of Sciences collaborate with the University of Chinese Academy of Sciences. The top 10 institutions with the most publications are listed in **Table 2**, with the University of Copenhagen coming in first with 134, followed by the Universities of Gothenburg and Helsinki. The University of Helsinki has the highest centrality (0.23), making it the most collaborative university.

**Table 2:** Top 10 institutions contributing to the number of articles.

Ranking	Institution	Centrality	Year	Publications
1	Univ Copenhagen	0.09	2011	134
2	Univ Gothenburg	0.04	2012	71
3	Univ Helsinki	0.23	2011	67
4	Catholic Univ Louvain	0.02	2011	65
5	Chinese Acad Sci	0.06	2012	63
6	Shanghai Jiao Tong Univ	0.00	2014	62
7	Harvard Med Sch	0.03	2016	60
8	Zhejiang Univ	0.01	2016	54
9	Univ Toronto	0.06	2011	48
10	Univ Turku	0.18	2011	47

### Main author contributions

**Figure 4:**
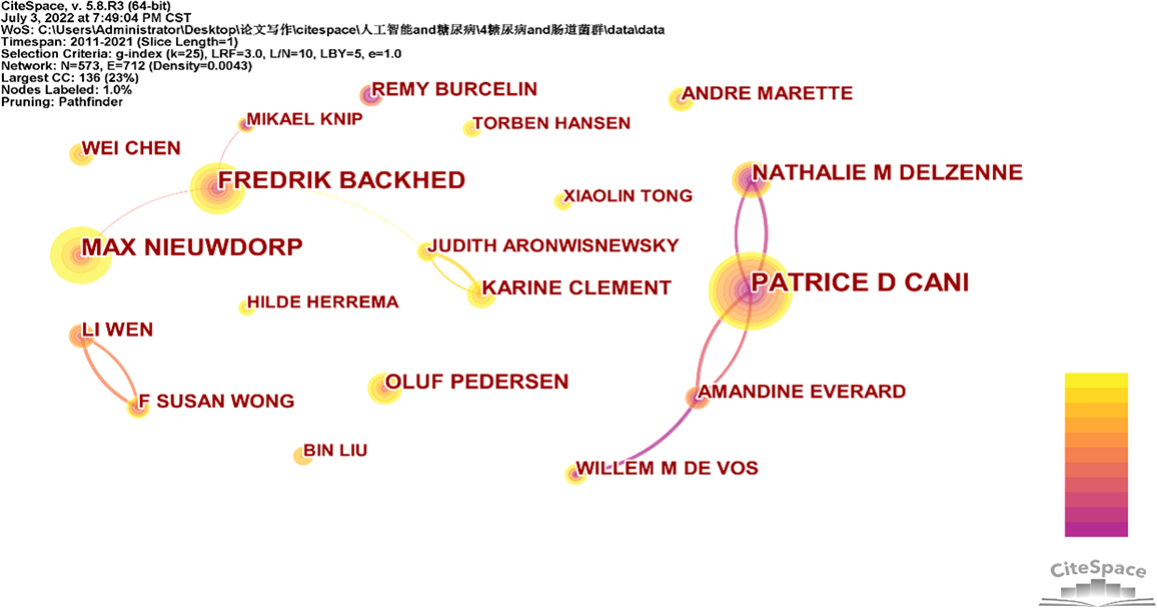
Collaboration between eminent article writers.

**Figure 4** depicts the network of cooperation between authors who have written 10 or more publications, and there are 23365 authors of research on diabetes and intestinal flora from 2011 to 2021. Along with LI WEN and F SUSAN WONG, PATRICE D CANI and AMANDINE EVERARD have also worked together. MAX NIEUWDORP and FREDRIK BACKHED have also cooperated. With 52 papers, PATRICE D CANI is rated first among the 10 authors in **Table 3** who have the most publications in this area. The centrality of ten writers is low, with Fredrik Backhed having the highest centrality (0.07).

**Table 3:** Top 10 authors with the highest number of articles.

Ranking	Author	Centrality	Year	Publications
1	PATRICE D CANI	0.03	2011	52
2	MAX NIEUWDORP	0.02	2015	38
3	FREDRIK BACKHED	0.07	2012	37
4	NATHALIE M DELZENNE	0.00	2011	25
5	OLUF PEDERSEN	0.02	2012	22
6	KARINE CLEMENT	0.04	2014	19
7	LI WEN	0.00	2015	17
8	AMANDINE EVERARD	0.04	2011	16
9	ANDRE MARETTE	0.02	2015	16
10	WILLEM M DE VOS	0.01	2011	15

### Keywords analysis

**Figure 5:**
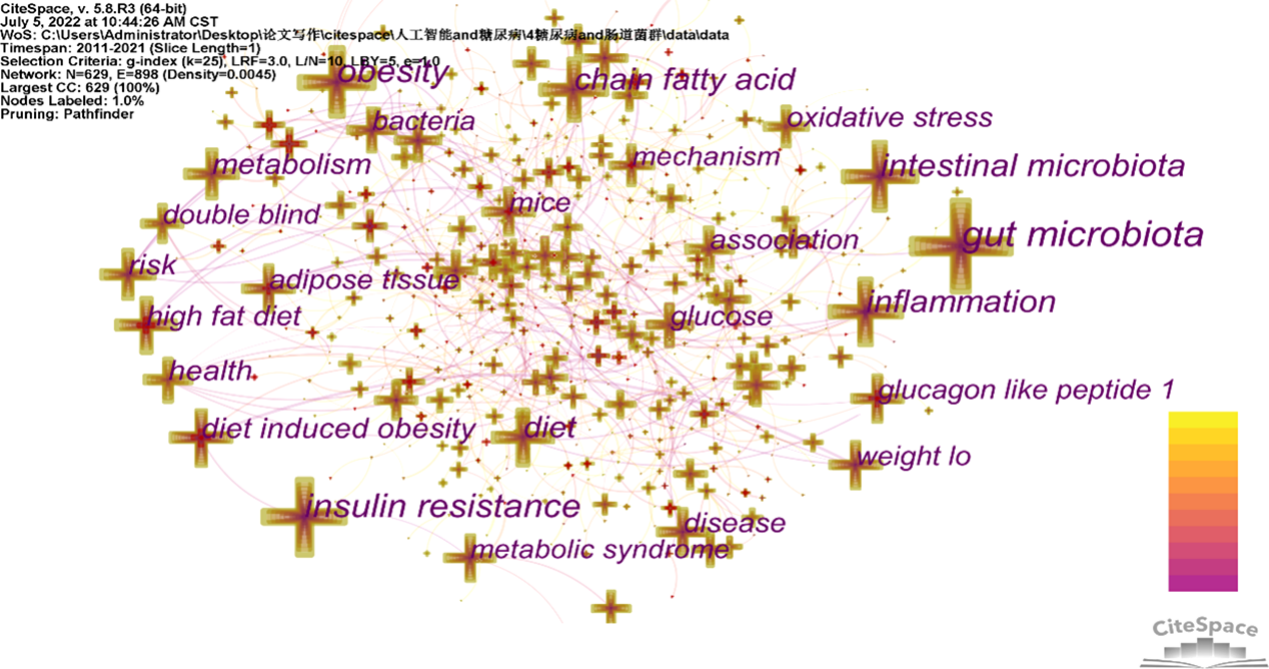
Visualization of keyword co-occurrence.

**Figure 6:**
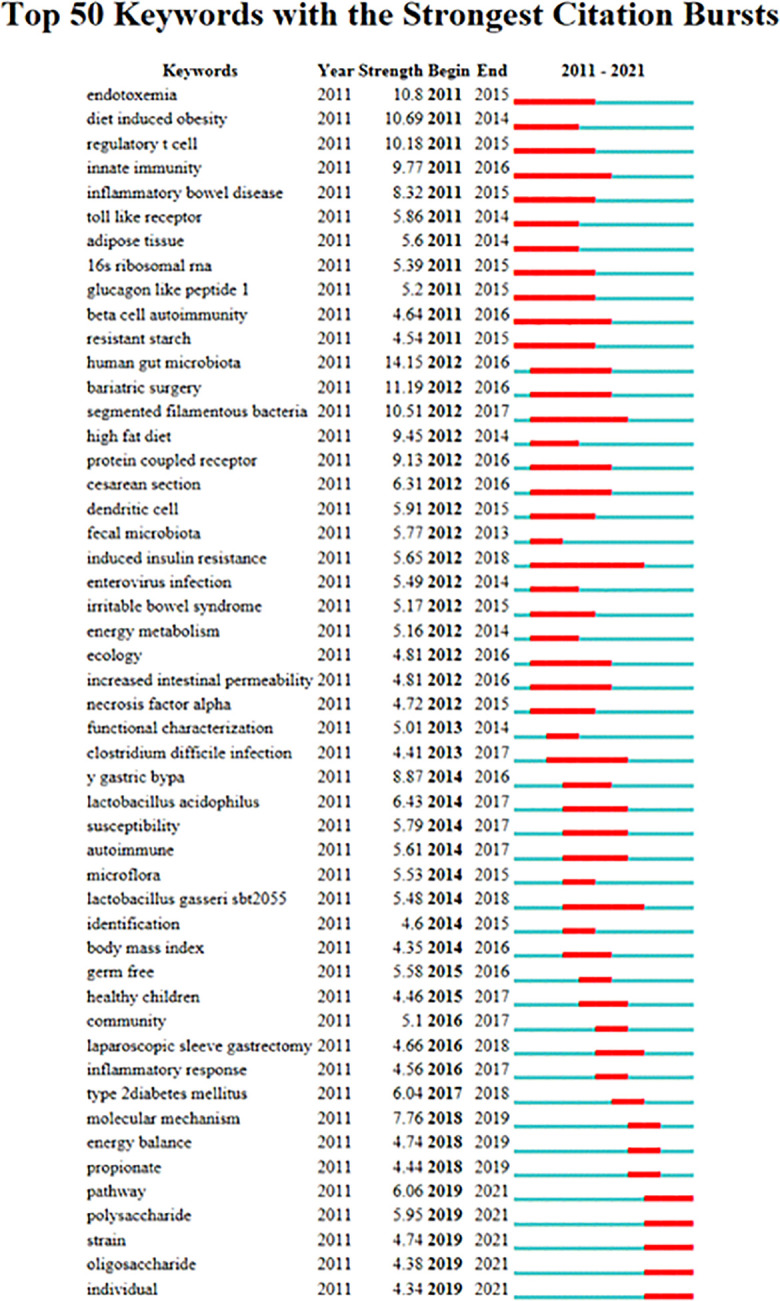
Visualization of Keyword Bursts.

The 4834 articles included in this analysis had 6698 keywords, and **Table 4** lists the top 20 terms with the highest frequencies. In terms of frequency, the phrase “gut microbiota” came in first place with a score of 1492, followed by the words “insulin resistance” and “fat.” The co-occurrence network of terms with a frequency exceeding 200 is displayed in **Figure 5**. **Figure 6** displays the top 50 terms in this field with the most intense outbreaks. Keywords like “diet-induced obesity” and “regulatory t cell” have outbreak intensities exceeding 10. Keywords like “pathway,” “polysaccharide,” “strain,” “oligosaccharide,” and “individual” have surfaced in the previous three years.

**Table 4:** The 20 keywords with the highest frequency.

Ranking	Keywords	Centrality	Year	Count
1	gut microbiota	0.00	2011	1492
2	insulin resistance	0.03	2011	774
3	obesity	0.04	2011	736
4	intestinal microbiota	0.01	2011	646
5	inflammation	0.04	2011	571
6	chain fatty acid	0.05	2011	468
7	diet	0.01	2011	420
8	metabolism	0.00	2011	328
9	diet induced obesity	0.04	2011	313
10	risk	0.06	2011	294
11	health	0.07	2012	286
12	high fat diet	0.00	2011	270
13	disease	0.00	2011	267
14	association	0.00	2013	260
15	glucose	0.12	2011	256
16	adipose tissue	0.00	2011	256
17	oxidative stress	0.01	2014	247
18	mice	0.01	2011	242
19	weight lo	0.05	2011	230
20	double blind	0.02	2012	229

### Analysis of high-yielding journals

**Figure 7:**
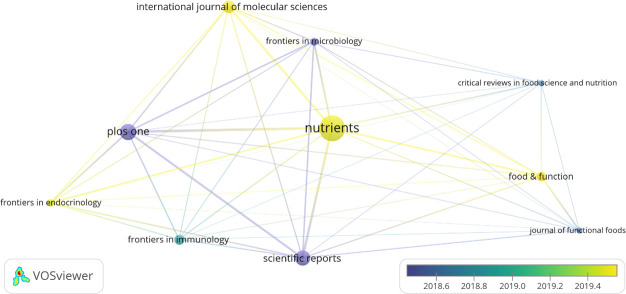
High-yielding journal visualization.

From 2011 to 2021, 4834 publications on research linked to gut flora and diabetes were published in 1256 journals, 40 of which contained at least 20 articles. **Table 5** lists the ten journals with the most publications. Nutrients, plos One, and scientific reports are the journals with more than 100 articles each. **Figure 7** depicts the link between the journals with the most articles and time, and the journals with active articles at the moment are international journal of molecular sciences, nutrition, diet & function, and frontiers in endocrinology.

**Table 5:** Top 10 journals with the highest number of articles.

Ranking	Journal	lmpact factor	citations	Publications
1	nutrients	6.706	7152	177
2	plos one	3.752	4903	111
3	scientific reports	4.996	3406	101
4	international journal of molecular sciences	6.208	2182	83
5	frontiers in immunology	8.786	1923	68
6	food & function	6.317	1585	66
7	frontiers in microbiology	6.064	2606	55
8	frontiers in endocrinology	6.055	900	48
9	critical reviews in food science and nutrition	11.208	1199	43
10	journal of functional foods	5.223	797	43

### Analysis of highly cited literature

The ten articles with the most citations are included in **Table 6**, along with three papers that have received more than 2000 citations. Functional interactions between the gut microbiota and the gut microbiota in type 2 diabetes was reported by Tremaroli et al. (11) in Nature and has 3491 citations. “Functional interactions between the gut microbiota and host metabolism” was written by Everard et al. (12), and “Cross-talk between Akkermansia muciniphila and intestinal epithelium controls diet-induced obesity” was published in Proceedings of the National Academy of Sciences of the United States of America.

**Table 6:** 10 highly cited literature.

Title	Journals	First author	Year	Citations
A metagenome-wide association study of gut microbiota in type 2 diabetes	Nature	Qin	2012	3491
Functional interactions between the gut microbiota and host metabolism	Nature	Tremaroli	2012	2548
Cross-talk between akkermansia muciniphila and intestinal epithelium controls diet-induced obesity	Proceedings of the national academy of sciences of the united states of america	Everard	2013	2285
Transfer of intestinal microbiota from lean donors increases insulin sensitivity in individuals with metabolic syndrome	Gastroenterology	Vrieze	2012	1612
Gut metagenome in european women with normal, impaired and diabetic glucose control	Nature	Karlsson	2013	1562
Short-chain fatty acids stimulate glucagon-like peptide-1 secretion *via* the g-protein–coupled receptor ffar2	Diabetes	Tolhurst	2012	1136
Personalized nutrition by prediction of glycemic responses	Cell	Zeevi	2015	1093
Sex differences in the gut microbiome drive hormone-dependent regulation of autoimmunity	Science	Markle	2013	1072
Alterations of the human gut microbiome in liver cirrhosis	Nature	Qin	2014	1060
The role of the gut microbiota in nutrition and health	Nature reviews gastroenterology & hepatology	Flint	2012	1053

## Discussion

This work is the first bibliometric evaluation of research on diabetes and intestinal flora, offering crucial data on this field of study to upcoming researchers. From the Web of Science Core Collection database, 4834 articles were gathered.

We started by looking at the publishing trend. Between 2011 and 2021, more articles on diabetes and gut flora were published each year. After 2019, there was a noticeable surge in publications. This could be connected to the rise in research on gut flora and polysaccharides in diabetes (13). The rise in publications suggests that more scientists are working on projects involving gut flora and diabetes. In 2011, Everard et al. (14) conducted research on how gut flora control helps diabetic mice’s blood glucose levels.

The United States has published 1262 publications during the past 11 years, which is the most of any nation for research on diabetes and gut flora. The United States, which has the most developed economy in the world, also funds the most research on gut flora and diabetes. The University of Copenhagen, which is located in Copenhagen, the capital of Denmark, is the organization with the most publications. This facility has demonstrated that controlling gut flora can stop type 2 diabetes (15). Patricia D. Cani is the author with the most publications, and her research suggests that changes in gut flora may contribute to diabetic inflammation (16). Patients with prodromal diabetes have abnormalities in their gut flora (16). Dysosmobacter welbionis, a human commensal bacteria, was shown to be able to prevent diet-induced obesity and metabolic problems (17). Nutrients is the most widely read publication, with 177 articles on gut flora and diabetes. In type 1 diabetes, nutritional variables play a significant role in controlling the gut flora (18). In diabetic individuals, mung bean seed coat extract can modify the gut flora (19).

Short-chain fatty acids (SCFAs), organic fatty acids produced by bacterial fermentation of large fibrous material in the distal gut, may improve characteristics of type 2 diabetes (20). Their main beneficial activities lie in reducing serum glucose levels, insulin resistance and inflammation, and increasing protective glucagon-like peptide-1 (GLP-1) secretion.Liu et al. (21) investigated how proanthocyanidins may alter intestinal flora to improve insulin resistance in gestational diabetes, which is a crucial aspect of the research of intestinal flora in diabetes. Proanthocyanidins from peanut peels can control gut flora to reduce type 2 diabetes’s insulin resistance (22). Dietary changes that alter gut flora can reduce insulin resistance (23). In-demand right now is the subject of pathways. Wu et al. (24)’s study on the effects of rhubarb tea extract on metabolic syndrome found that it reduced adipogenesis and altered microbiota through the SIRT6/SREBP1 pathway. Urolithin A was investigated by xiao et al. (25) to treat intestinal barrier malfunction and cognitive impairment brought on by diabetes. Dioscorea and Cornus officinalis were found by chen et al. (26) to reduce testicular damage in diabetic rats *via* the butyric acid/glucagon-like peptide-1/glucagon-like peptide-1 receptor pathway, which is mediated by the intestinal microbiota. In recent years, polysaccharides have also played a significant role in research. Pumpkin polysaccharides can alter the flora in type 2 diabetics’ digestive tracts (27). In type 2 diabetes, ganoderma lucidum polysaccharides can control the flora in the gut (28). Polysaccharides increase the amount of Lactobacillus that controls the flora in the digestive tract (29). In addition, bariatric surgery affects the gut flora of diabetes patients (30, 31). There will be more information regarding diabetes and gut flora as research advances.

### Strengths and limitations

The bibliometric evaluation of works on diabetes and intestinal flora is presented in this paper for the first time. Our bibliometric study was more thorough and clear than the literature review because we employed a systematic search and quantitative statistical analysis. Our study does have certain shortcomings, though. Although the great majority of articles are in the Web of Science Core Collection database, the information might not be full.

## Conclusion

In this study, Citespace.5.8.R3 and Vosviewer1.6.17 were used to evaluate 4834 publications on studies relating diabetes and gut flora from 2011 to 2021. Over the past 11 years, research on diabetes and gut flora has grown, most significantly in the last 3 years. Research in this field is being conducted by 23365 authors in 109 nations and 4820 institutions. This bibliometric analysis serves as a resource for scholars.

## Data availability statement

The original contributions presented in the study are included in the article/**Supplementary Material**. Further inquiries can be directed to the corresponding authors.

## Author contributions

The data were compiled by SX and FJ, the manuscript was written by LZ, it was reviewed by HZ and FZ, and it was financially supported by FY, JG, YD, BH, and CW. KX, HZ, QX and FY revised the manuscript. All authors contributed to the article and approved the submitted version.

## Funding

This research was funded by National Natural Science Foundation of China (82174236), Hospital of Chengdu University of Traditional Chinese Medicine (19PJ04), Sichuan Administration of Traditional Chinese Medicine (CKY2021106), Jiangxi Provincial Natural Science Foundation Youth Fund (20202BAL216065), Jiangxi Provincial Education Department Science Program (GJJ201259), Jiangxi Provincial Science and Technology Department (20212BAG70037), Sichuan Provincial Department of Science and Technology(2021YFS0268), Sichuan Provincial Department of Science and Technology (2022JDKP0082), Sichuan Provincial Department of Finance (CJJ2022055).

## Acknowledgments

For supplying the original data for this work, we are grateful to the web of science core collection.

## Conflict of interest

The authors declare that the research was conducted in the absence of any commercial or financial relationships that could be construed as a potential conflict of interest.

## Publisher’s note

All claims expressed in this article are solely those of the authors and do not necessarily represent those of their affiliated organizations, or those of the publisher, the editors and the reviewers. Any product that may be evaluated in this article, or claim that may be made by its manufacturer, is not guaranteed or endorsed by the publisher.
